# Initiating Cognitive Processing Therapy (CPT) in Community Settings: A Qualitative Investigation of Therapist Decision-Making

**DOI:** 10.1007/s10488-022-01229-8

**Published:** 2022-11-12

**Authors:** Fiona C. Thomas, Taylor Loskot, Christina Mutschler, Jessica Burdo, Jansey Lagdamen, Iris Sijercic, Jeanine E. M. Lane, Rachel E. Liebman, Erin P. Finley, Candice M. Monson, Shannon Wiltsey-Stirman

**Affiliations:** 1 Department of Psychology, Toronto Metropolitan University, Toronto, Canada; 2 Department of Psychiatry, US VA National Center for PTSD Dissemination and Training Division, Stanford School of Medicine, Stanford, USA; 3 Ontario Shores Centre for Mental Health Sciences, Whitby, Canada; 4 Department of Psychiatry, University Health Network, Toronto, Canada; 5 Departments of Medicine and Psychiatry, UT Health San Antonio, San Antonio, TX USA; 6 VA Greater Los Angeles Healthcare System, Los Angeles, CA USA

**Keywords:** Cognitive processing therapy, Community providers, Qualitative analysis, Decision-making, Implementation, Community mental health

## Abstract

Various organizations have provided treatment guidelines intended to aid therapists in deciding how to treat posttraumatic stress disorder (PTSD). Yet evidence-based psychotherapies (EBPs) for PTSD in the community may be difficult to obtain. Although strides have been made to implement EBPs for PTSD in institutional settings such as the United States Veterans Affairs, community uptake remains low. Factors surrounding clients’ decisions to enroll in EBPs have been identified in some settings; however less is known regarding trained therapists’ decisions related to offering trauma-focused therapies or alternative treatment options. Thus, the aim of the current study was to examine therapist motivations to initiate CPT in community settings. The present study utilizes data from a larger investigation aiming to support the sustained implementation of Cognitive Processing Therapy (CPT) in community mental health treatment settings. Enrolled therapists participated in phone interviews discussing their opinions of CPT, preferred treatments for PTSD, and process in assessing appropriate PTSD treatments for clients. Semi-structured interviews (*N* = 29) were transcribed and analyzed using a directed content analysis approach. Several themes emerged regarding therapists’ decision-making in selecting PTSD treatments. Therapist motivations to use EBPs for PTSD, primarily CPT, were identified at the client (e.g., perceived compatibility with client-level characteristics), therapist (e.g., time limitations), and clinic levels (e.g., leadership support). The results provide insight into the complex array of factors that affect sustainability of EBPs for PTSD in community settings and inform future dissemination of EBPs, including training efforts in community settings.

Posttraumatic stress disorder (PTSD) is a mental health condition that is associated with myriad individual and societal costs (Kessler, 2000; Van Ameringen et al., 2008). Untreated and chronic PTSD is associated with suicidality, substance use, psychosocial problems, and a range of other issues (Jakupcak et al., 2009; Osei-Bonsu et al., 2017; Sayer et al., 2010) that make it essential to determine ways for individuals with PTSD to have access to effective treatment. Though a variety of efficacious evidence-based psychotherapies (EBPs) for PTSD have been developed, these treatments are underutilized in community settings, such as in private practice settings, community hospitals, and outpatient mental health clinics (Finley et al., 2019). Cognitive Processing Therapy (CPT; Resick et al., 2016) is one such first-line recommended intervention for individuals with PTSD (e.g., American Psychological Association, 2017; Forbes et al., 2020; Veterans Health Administration & Department of Defense, 2017). CPT is a manualized, time-limited, trauma-focused treatment. The intervention consists of 12, 60-min sessions that incorporate cognitive restructuring techniques along with emotional processing of trauma-related memories and beliefs (Resick et al., 2016). Despite current clinical practice guidelines recommending CPT as first-line intervention for PTSD, psychotherapy providers typically determine the course of treatment for the individuals with whom they work, and may not offer EBPs to all of their clients (Cook et al., 2014; Finley et al., 2019).

Research suggests there are many factors involved in the adoption and delivery of EBPs generally, which may be a combination of therapist, client, and organizational factors (Aarons et al., 2011; Asgary-Eden & Lee, 2012; Moore, 2002). Factors identified in the literature include therapist characteristics (e.g., attitudes toward EBPs or demographics), client concerns regarding stigma or negative perceptions of mental healthcare, and variables at the organizational level such as infrastructure support, strong leadership, and organizational climate (Osei-Bonsu et al., 2017; Sijercic et al., 2020). To date, client and organizational factors have received significant attention in the literature (e.g., Aarons & Sawitzky, 2006; Hundt et al., 2018; Maguen et al., 2019); however, fewer studies have examined motivations for utilization directly from therapists’ perspectives. Relatedly, there remains limited understanding of what influences community providers’ decision to engage in trauma-focused EBPs. Clinicians in community settings do not always have the same access to resources, consultation, or training as their counterparts in institutional settings, which may result in delivering care of variable quality (Finley et al., 2019). At the same time, many individuals access care in community settings as their first point of contact, making community-based clinicians an essential population to target for EBP dissemination efforts. Before dissemination, however, it is imperative to first understand existing practices. As such, the aim of the current study is to qualitatively examine therapist motivations to initiate CPT in community settings.

Emerging literature on the process of provider decision-making for implementation of specific PTSD interventions exists (e.g., Finley et al., 2020; Garcia et al., 2019); however, much of this literature is from the perspective of therapists in institutional settings (e.g., publicly-funded healthcare systems such as Veterans Affairs). Although research on therapists’ perspectives implementing CPT specifically is limited, several recent studies conducted in VA settings have examined therapists’ viewpoints regarding the utilization of EBPs for PTSD. Literature has identified several reasons that influence therapist decision-making to implement EBPs with their PTSD clients. First, client complexity is an often-cited rationale for why therapists opt not to implement a PTSD-targeted EBP with their clients. For instance, Osei-Bonsu et al. (2017) conducted qualitative interviews with therapists in VA medical centres working with individuals with PTSD to better understand their decision-making related to initiating CPT or Prolonged Exposure (PE; Foa et al., 2007). The authors found that client comorbidity (i.e., concurrent substance use disorder or personality disorders) influenced their decision-making around whether to initiate these treatments. Similarly, Cook et al. (2014) conducted qualitative interviews with therapists in the VA to better understand client factors that guided their decision to initiate CPT or PE. They found that both the presence of comorbidities that required stabilization or that could limit an individual’s ability to benefit from PE or CPT, and cognitive limitations, influenced therapists’ decisions of whether to initiate CPT or PE. Lu et al. (2016) likewise identified that clinical considerations, such as client crisis or skill building before trauma-focused treatment, and veterans’ beliefs, stigma, or lack of engagement, influenced therapist decision-making. Secondly, therapist perceptions of client readiness and motivation (e.g., willingness to engage, having adequate coping skills and stability) is another often-cited reason in the literature to not implement an EBP for PTSD (e.g., Cook et al., 2014; Osei-Bonsu et al., 2017). In another qualitative study that examined therapist chart notes in VA settings, client access barriers (e.g., geographic location, fees, practical barriers) and clinician barriers (e.g., therapist schedules, changing jobs) were found to be obstacles to implementing CPT (Lu et al., 2016). Although CPT treatment developers recommend only a limited list of contraindications, including imminent suicidality/homicidality, substance use disorder requiring medical detoxification in order to reduce use, and uncontrolled mania or psychosis (Resick et al, 2016), existing literature point to a wider variety of reasons why therapists may choose to not initiate an evidence-based, trauma-focused therapy.

Therapists in community settings tend to have less experience and training in EBPs for PTSD (Finley et al., 2019), making it likely that implementation rates of CPT are even lower in the community than in institutional settings (Hundt et al., 2018). Utilization of EBPs in community settings may remain low even after therapists have received training and consultation (Dondanville et al., 2021a; Finley et al., 2018). A recent survey study examined initiation rates of EBPs in community settings serving veterans following a training program for CPT and PE. CPT patient initiation rates ranged from 30 to 35% over a 5-month follow-up period (Dondanville et al., 2021a). In another study that examined implementation barriers of a community-based training program, half of the clinicians who received CPT training reported having implementation challenges including referral issues, patient disinterest in the treatment, and low confidence in delivery of CPT (Dondanville et al., 2021b). However, in light of low utilization rates, there are likely a variety of other reasons why therapists may not deliver CPT. Elucidating these factors would increase our understanding of organizational and system barriers that are unique to community settings.

Therapists in community settings report low suitability of trauma-focused EBPs for their clients, due to complexity of clients, including clients with multiple traumas and long-standing trauma history (Marques et al., 2016). One study found that therapists in community settings reported poorer job satisfaction, greater burnout, and higher likelihood of leaving their job compared to therapists in VA settings (Salyers et al., 2013), all factors found to be related to lower reach of EBPs (Glisson et al., 2008). Relatedly, organizational factors such as limited staffing and perceptions of not being part of a coherent team were additionally associated with fewer hours spent using individual CPT (Finley et al., 2015).

At the intersection of client and therapist is the role of shared decision-making, which implies full and collaborative participation of the provider and client in making treatment decisions (Drake et al., 2009). In mental health, research suggests that shared decision-making facilitates a strong working alliance, which contributes to improved long-term outcomes (Deegan & Drake, 2006; Gibson et al., 2020). Given the therapist, client and organizational factors noted above, it remains unclear how clinicians in community settings engage in shared decision-making with their clients regarding decisions to engage in trauma treatment.

Thus, the aim of the present study was to understand how therapists in community settings decide to initiate CPT with individuals with PTSD and to examine the factors that impact their decision-making, including the role of shared decision-making. The present study is embedded within an ongoing, longitudinal, randomized controlled implementation trial (Wiltsey-Stirman et al., 2017). By qualitatively examining the key factors related to implementation for community-based therapists trained in CPT, our objective is to expand the literature in this understudied area to ultimately support the delivery of high-quality care for PTSD in community settings.

## Method

### Ethics Approval

Ethics approval for the parent study was received from the Institutional Review Board at Toronto Metropolitan University in 2014 and at Stanford University and Palo Alto Veterans Healthcare Administration System in 2015. Additional approvals were sought and received from each individual partner organization.

### Sampling and Data Collection

Semi-structured interviews were conducted as part of the parent study (Wiltsey-Stirman et al., 2017). For the purposes of the current study, qualitative interview data from the baseline phase of the parent study was included for analysis. Specifically, participants included clinicians from community/hospital clinics and community-based practitioners across Canada and the United States. Sites included private practice settings (*n* = 16), community hospitals (*n* = 9), and community-based outpatient mental health clinics (*n* = 5). Therapists from these sites were eligible to participate in the study if they were providing psychotherapy to individuals with a diagnosis of PTSD. Thirty community-based therapist baseline interviews were conducted during the parent study, with the majority of clinicians identifying as female (80%). Level of training was approximately equally divided between the MA/MSc (30%), LCSW/MSW (23.3%) and PhD/Psy.D levels (23.3%). The majority of providers were based in Canada (86.7%). Please see Table 1 for participant demographics. Due to an audio-recording error with one interview, transcripts from 29 interviews were included in the current sample for analysis. Of note, although the focus of this study was on the non-military/Veteran population, some private practice therapists in our sample worked with the Veteran population. The unique circumstances of working with this population are discussed further in the Results section.

**Table 1:** Provider demographics (*N* = 30)

Sociodemographic characteristic	Canada *n* = *26*		US *n* = *4*		Full sample *N* = 30	
	*n*	*%*	*n*	*%*	*n*	*%*
Gender						
Female	22	84.6	2	50	24	80
Male	4	15.4	2	50	6	20
Age group						
20–29	3	11.5	1	25.0	4	13.3
30–39	10	38.5	1	25.0	11	36.7
40–49	3	11.5	2	50.0	5	16.7
50–59	7	27.0	0	0	7	23.3
60 years or older	3	11.5	0	0	3	10
Educational level						
PhD/PsyD	7	27.0	0	0	7	23.3
MD	3	11.5	0	0	3	10
MA/MSc	8	30.8	1	25.0	9	30
LCSW/MSW	4	15.4	1	25.0	5	16.7
Other	4	15.4	2	50.0	6	20

### Procedure

Over the course of the 5-year study, eight trained research assistants conducted the semi-structured interviews independently via phone or using a secure virtual platform (i.e., Zoom). Audio recordings of interviews were transcribed and de-identified by a HIPAA-compliant professional transcription service and then verified by research assistant interviewers.

#### Interview Guide

The semi-structured interview guide was initially developed based on an extensive literature review and was informed by the Consolidated Framework for Implementation Research (CFIR; Damschroder et al., 2009), with a focus on common barriers and facilitators to effective implementation. Additional questions were added to address emergent research questions, including the objectives for the current study. The interview covered key domains including: therapist experiences with EBPs, particularly CPT and its comparison to other modalities; perceived client response to CPT and EBPs generally; and therapists’ perception of their workplace and prioritization of EBPs.

### Data Analysis

Data analysis was conducted with interview transcripts using directed content analysis (Hsieh & Shannon, 2005). This approach involves the application of previous research and theory in order to inform the initial coding schemes and the development of themes. For the present study, the coding framework was developed and finalized for a prior study exploring similar research questions, with a Veteran-serving subsample of clinicians from the same parent study (see Loskot et al., 2022). The same coding framework was applied in the current study, with revisions to capture additional themes that were unique to the current sample of community-based clinicians. The coding framework was refined using existing literature as well as emerging themes within the data analysis.

Data was coded and analyzed with Dedoose, a qualitative software management program (Dedoose Version 9.0.17). Four researchers were involved in the coding process (CM, FCT, JB, and TL). First, each researcher independently coded two transcripts using the coding framework developed by Loskot et al. (2022). The coding team then discussed how their coding aligned or diverged across the two transcripts. Additional revisions were made to the coding framework based on analysis of themes and clarifying descriptions of codes. Discrepancies were discussed until consensus was reached. The process of calibration continued until no further changes were made to the coding framework and the divergences across coding were minimal.

After calibration was complete, the four researchers independently coded the remaining transcripts. To reduce conceptual drift, the coding framework included definitions for each code, along with examples from the literature and initial coded transcripts. Further, 25% of transcripts were double-coded by another member of the coding team in order to ensure intercoder reliability (O’Connor et al., 2020). Lastly, the transcripts coded during the calibration process were re-coded based on the final coding framework.

Throughout the coding and analysis process, bias and personal reflexivity were continuously reviewed. Inherent in directed content analysis approaches is a bias to the existing literature and theory that drives the initial coding. However, as codes that did not fit into the codebook were identified, the team met and discussed appropriate codes to better fit the data or create new codes as appropriate. This process was documented so as to name biases and remain neutral to coding and analysis, a best practice within directed content approaches (Hsieh & Shannon, 2005).

Once all transcripts were coded, code reports were created. Coded data extracts were sorted based on the broad categories in the coding framework (i.e., clinician, system, treatment, other). The extracted data were divided amongst the coding team to review emerging themes. By reviewing the multiple codes and corresponding excerpts within the broad categories, each team member could delve further into identifying the relationship between codes and their importance in relation to the overall research questions (Braun & Clarke, 2006). To initially identify intersecting themes, a coding report from Dedoose was produced that highlighted the most frequently co-occurring codes. Using this as an initial starting point, iterative discussions between members of the coding team further facilitated the examination of intersecting themes. This approach allowed for the relationship between emerging themes to be explored and documented. Thus, the focus was on the *significance,* not the frequency of a code, thereby ensuring that coding was focused on understanding how particular themes connected to others (Creswell & Creswell, 2017).

A mix of inductive and deductive analyses were applied. As noted, the interview guide was informed by the CFIR framework and literature in the area. Subsequently, the coding framework included constructs relevant to CFIR and implementation science, more broadly. In this way, the researchers applied a deductive approach where analysis of codes and excerpts were informed by a preexisting coding framework and theoretical knowledge of the literature. At the same time, inductive analysis was also applied in tandem where data-driven codes were included even if they did not fit within a pre-existing category of the framework. This flexible analytical approach allowed the research team to identify how results from the current study complemented existing literature while highlighting novel themes. This approach is documented in the implementation science literature where interview questions and the coding framework may be informed by a particular theory, but emergent themes are identified to reflect participant experiences (e.g., Sayer et al., 2017).

As codes were collated and themes and subthemes were identified, the research team met regularly to review emerging themes and the relationship between themes. By reviewing emerging themes together, the team was able to determine coherent patterns and their relationship to the overall research question (Nowell et al., 2017). Themes were discussed and vetted with the broader research team to ensure reflexivity in the process. Once themes were identified, time was spent defining and naming themes and discussing how each theme fit with the research questions. The research team subsequently organized the themes until consensus was reached, and the team felt that all data were meaningfully represented (Nowell et al., 2017). Finally, four uncoded transcripts were reviewed by individual members of the coding team. The emerging themes were tested using these transcripts in order to assess for saturation. During this phase, no new themes emerged nor did any transcripts deviate from the existing framework, providing further evidence for data saturation and confirmation of themes.

## Results

Inductive and deductive thematic analysis provided the basis for themes, which primarily emerged at the system/clinic and clinician levels. Our findings revealed further nuanced relations between providers’ decisions to implement CPT, system-level factors that can facilitate or impede implementation, and perception of client-level characteristics that may influence clinicians’ decision to implement CPT. Themes are presented in alignment with the domains of CFIR, including outer and inner setting, characteristics of individuals (i.e., patient and provider), and characteristics of the intervention. The outer setting refers to factors in the external system of the intervention, while the inner setting refers to factors internal to the organization itself. Intersecting themes within these domains were also mentioned by clinicians. We present exemplar quotes to highlight these emergent themes.

### Outer Setting

Providers identified system-level factors that influence their decision to implement CPT, including the role of insurance and the referral system.

#### Policies and Incentives-Funding for Treatment and the Role of Insurance

Community-level providers described two main issues that impacted their ability to implement CPT in their respective settings. First, internal and external policies either encouraged or discouraged the implementation of CPT. More specifically, providers who worked in publicly-funded healthcare systems noted that external (i.e., government) funding was helpful for increasing access to services for clients in some cases, and posed barriers in others. In community settings serving the military/veteran population for instance, providers noted that government funding resulted in additional administrative hoops to navigate; however, this was not necessarily a barrier to implementing CPT with their clients. As noted by one provider,

Here in [location], things that are paid for by the government, it tends to be burdensome. Like this morning I had to spend excessive amounts of time trying to...go through a foolish government system…[But agency] is extremely generous, [agency], as well. If you're running out of sessions, you write a report and they top you up and give you the sessions you need...They just have a bit of a convoluted system so sometimes you're...calling all over to get a bill paid but in terms of limiting my number of sessions, no, I don't think that's a factor at all (Clinician, private practice).

In other publicly-funded settings however (e.g., community hospitals), providers reported challenges with implementing CPT when the number of sessions were limited or clients were faced with long waitlists. As highlighted by the clinician below,

…psychotherapy services offered in hospitals are covered by [insurance program], but again, it's very, very time limited. And unfortunately, psychotherapy in Canada, especially Ontario, you'd have to pay out of pocket [in private practice settings]. And I think it's a shame because there's so many clients who need it. And many of them are on financial assistance and government assistance, and they can't afford the therapy. So, we try to make it available in general hospitals...again, there's this restriction of what is able to be covered….the ones that are [insurance program] covered have like a 2-year long waitlist (Clinician, provincial health agency).

Similar to providers in community-hospital settings, providers in private practice settings were often navigating private insurance policies, which imposed limitations on the number of treatment sessions clients could access due to limited funding availability. As noted by the provider below, limited financial resources from the client resulted in whether or not they offered CPT or modified its implementation dose:

When people are on third-party providers here like [agency] and [agency], it's all fine and actually it's ideal because they get lots of sessions. And they prefer that you use evidence-based treatments so it's easy to justify, however when people are paying privately...most people have maybe $500–$1000 coverage, which does not go very far. And so that's also a decision that I'll make if I think a person's only got five or six sessions that they can afford, that's when I'll be selective (Clinician, private practice).

Together, these hurdles either limited provider decisions to implement CPT, or prompted them to modify the way sessions were delivered.

#### Referral Source and Advocacy

Although not explicitly listed in the CFIR, the way in which people were referred to treatment, and the appropriateness of those referrals impacted provider decisions. Providers described receiving referrals from various sources, including insurance providers, employee assistance programs (EAPs), other community-providers (e.g., case managers) or clinicians (e.g., family physicians) from other settings. Thus, although referrals were described as mostly appropriate for CPT in community settings, some providers noted that when clients were assessed and diagnosed with PTSD externally (e.g., by a provider in another setting), it was not always clear how external parties determined PTSD diagnosis or fit for CPT. Occasionally, this resulted in inappropriate referrals and the need to reassess and/or discontinue CPT engagement. When it came to private insurance referrals, some providers noted the common misconception of insurance companies that trauma-focused therapy is inevitably long-term and thus the need to dispel some of these myths. The variability in referral sources resulted in some clinicians highlighting the need for advocacy efforts and general education for healthcare providers regarding CPT to facilitate appropriate referrals. The following interviewee’s statement typified this sentiment:

...we're doing a presentation to one of our referral sources, an EAP [Employee Assistance Program], on Friday about our use of this therapy [CPT] for trauma and one of my slides is called "The Beauty of CPT" and it's about how it is effective...That it's nice and short, it's doable for people. And recognizing that a lot of other therapies work, but this one works fairly quickly and it gives people the skills to continue to, I guess, be their own therapist (Clinician, private practice).

### Inner Setting

Providers discussed factors within the organizations in which they were situated that impacted their decisions to initiate CPT. These included leadership support and time limitations.

#### Leadership Support

Overall, providers in the current sample expressed a positive perception of leadership in their respective organizations. Across the various settings, leaders were described as being supportive of EBP implementation. As highlighted by one provider, “I think they've gone towards these EBTs [evidence-based treatments] and… I would suggest management has gone out of their way to make sure everyone gets trained in whatever protocol they're using” (Clinician, provincial health agency). In private practice settings in particular, providers noted the benefits of autonomy and flexibility in their work. As another clinician described, “I'd say the freedom to make your own hours. You get to really pick the populations you want to work with, which is really cool…you get to set up shop, and do what you only want to do versus if you worked for maybe a government run organization, you're going to have to do whatever's given to you. The freedom is nice” (Clinician, private practice).

Relatedly, private practice providers endorsed feeling well-supported to attend CPT workshops but noted that there were not always incentives to do so. For example, private practice clinicians noted that they attended CPT training on their own accord and expense and additionally lost income by not seeing clients on days when they attended workshops; however, these factors were not entirely a deterrent in learning CPT for the subset of providers enrolled in this study.

#### Available Resources: Time Limitations

To a lesser extent, some providers noted that limited time available functioned as a barrier to implementing CPT. In particular, demands within the workplace, managing high caseloads, and limited staffing were noted as factors that interfered with CPT implementation. As one provider shared, “I would need to have fewer clients, really… I think that's a big issue. The demand is so big, and you just try to accommodate as much as you can, but it's hard. It's a lack of time, honestly” (Clinician, private practice). Although some providers noted limited time as a barrier to engaging in CPT, this finding did not emerge for the majority of clinicians in the current study.

### Provider- and Client-Level Factors

Providers identified several factors based on their own personal attributes, training background, and sense of self-efficacy that prompted them to learn and implement CPT. Relatedly, they described client-level factors that influence their decision of whether or not to implement CPT, as well as their approach to engaging clients in CPT.

#### Provider-Level Characteristics

Overall, providers expressed a positive view of CPT, and many indicated that the treatment approach aligned well with past training in Cognitive-Behavioral Therapy (CBT). Novice and seasoned providers described the structured approach as helpful, and appreciated the evidence-based perspective. As noted by one provider,

I love it. I've definitely seen clients improve from it. I think that I definitely have a buy-in because I've seen that change. And I like the way that it's laid out in terms of the sessions and the way that they build [on each other]. I think it's perfect. And then, again, as a new therapist, I think it's easy to follow. It's intuitive. And I think the more practice that I get, the better I've been getting at it, like I see improvement. (Clinician, community hospital).

Interestingly, a minority of providers in this sample described their initial skepticism of CPT, particularly those who were not previously trained in manualized approaches. These providers subsequently highlighted their shift in perspective after implementing CPT and seeing symptom improvement in their patients.

I'm not someone who has had a lot of experience with using a treatment protocol in the way that CPT is so set out, so manualized, I guess. I'm not used to that. Really was resistant to the idea and very much thinking you couldn't do that kind of treatment and be really individualized at the same time, which is something I've discovered is not true. So, it's been a really good experience for me in that way too, because I'm able to take that manualized approach, use it quite effectively...And that's the thing that struck me most with this therapy is that, for 80 percent of the people I've worked with using it, it's been really, really helpful (Clinician, private practice).

#### Time to Develop Comfort with Implementing CPT

Aligned with findings at the system level where providers described the limitations of time because of administrative barriers, a minority of providers in this sample alluded to the time it takes to learn and prepare for CPT sessions. Interestingly however, these providers did not indicate that CPT learning or preparation time was a barrier to implementation. Rather, they described feeling more proficient over time with increasing familiarity and engagement with the protocol. As this private practice clinician stated, “it could take some front-end work, so if you're willing to put some front-end time into it, I think it will pay off in the long run and then you kind of just hone in your skills over time” (Clinician, private practice).

#### Patient Needs and Resources: Engaging Clients in CPT

Some providers described the importance of client choice in therapy options. One provider explained, “I find that having options is really the best for them [clients] because, again, it involves them, they understand more about what the differences in the approaches are and then they can choose what seems to fit best for them” (Clinician, private practice).

Therapy options offered were based on what therapists were trained in and felt comfortable implementing. However, in line with trauma-informed care, therapy choices were offered in efforts to empower clients to make their own decisions and promote control and choice in therapy. In this way, client choice of therapy was prioritized for effective therapeutic engagement.

### Characteristics of the Intervention

#### Perceived Compatibility

Beyond a definitive diagnosis of PTSD, providers described several other client-level factors that prompted their decision of whether to engage in CPT. Most often, it appeared that the decision to implement CPT with a particular client was based on clinical judgement, rather than literature-informed decisions regarding who may be a good fit for CPT. Providers noted that they were more likely to implement CPT based on the following client characteristics: personal motivation; cognitive flexibility; stability (e.g., housing, social support system); higher education levels; stable employment; single trauma; or type of trauma (e.g., sexual assault). Providers noted that they were *less likely* to deliver CPT based on client complexity (e.g., psychosocial challenges like housing issues), significant comorbidities (especially, active substance use disorder or personality disorders), or if they presented with multiple traumas or a long-standing trauma history (e.g., childhood sexual abuse). As stated by one provider:

The more stable the basic needs of the individual are, the better. So, someone not having troubles with shelter or food. Because, trying to focus on therapy when they've got problems like that, is a problem. Anybody that is going through any significant life events...I think, people who are more focused and have less things going on. Definitely anybody that has a knowledge of coping, they are better suited for the model (Clinician, community clinic).

Clinicians noted the challenges of engaging clients with a long-standing trauma history in CPT because of their strong identification with their diagnosis and some resistance to change. When providers noted that they were less likely to use CPT for the reasons noted above, they described turning to a range of alternative options based on perceived client needs. For instance, some providers noted that they offered another trauma-based EBP (such as Prolonged Exposure if they felt the client was a better fit for behavioral interventions). In instances where clients needed further psychosocial support before engaging in CPT, providers noted that they initiated a stage-based approach, prioritizing psychosocial stability for complex clients and/or teaching coping skills before implementing CPT. However, the stage-based approach did not impact their decision to eventually engage in CPT once the client was stabilized; it delayed the start of the intervention. Despite some characteristics noted above as interfering with provider decisions to implement CPT, interviewees noted that generally clients responded positively to EBPs and CPT. Thus, client perception of EBPs did not appear to influence therapists’ decision to use CPT.

### Intersecting Factors

Additional themes emerged from the analysis of codes that commonly co-occurred together across system, provider, and client levels. These themes reflect intersecting factors (i.e., cross-level factors) that contribute to providers’ decision-making to implement CPT.

#### Shared Decision-Making Models

Codes that intersected at the provider and client levels highlighted a shared decision-making model regarding the implementation of CPT. Specifically, clinicians described a shared decision-making approach when initially introducing treatment options to their clients. This was coded as the clinician’s procedure for implementing CPT. The clinician’s procedure often included providing a description of CPT and other trauma-focused therapies (e.g., Prolonged Exposure). From the client level, codes in the data highlighted how clients had a say in their care, depending on their readiness and goals for treatment. Clients presented their personal preferences for therapy and desire to engage in CPT. Those who had psychosocial concerns and less stability, preferred treatment that did not include homework. Relatedly, individuals who presented with other mental health issues they wanted to address first, often opted for an alternative treatment approach. As exemplified by one private practice provider:

It’s a very positive discussion. If we go to any sort of practitioner, you want to be informed of the choices that you have. If you go to physicians, for example, well, I can give you this medication that has these side effects or I could give you this one, which is a little easier on your stomach whatever the case may be, right? So, being able to inform your clients that there are a number of different ways of treating trauma and this just happens to be one of the ones I'm familiar with like, for example, you could suggest EMDR but I don't do that but it is potentially a valuable treatment for some forms of disorder. So, I like the ability to give clients a choice (Clinician, private practice).

#### Fragmented Community Setting

Codes that intersected at the system and client levels indicated a fragmentation of care within community settings compared to institutional settings. This theme of ‘Fragmented Community Setting’ intersected at the system, clinic, and client level. Clinicians noted the challenges of engaging clients with long-standing trauma histories as well as significant psychosocial stressors. Clinicians described that institutional settings where patients can receive psychosocial support within the same system (i.e., more integrated approaches in institutional settings such as the VA) can assist with navigating a variety of these barriers. In contrast, clinicians in community settings often have to provide support for psychosocial stabilization before engaging in CPT, or need to refer out for stabilization before they can implement CPT. Often when providers noted that they were less likely to use CPT for the reasons noted above, they described turning to a range of alternative options based on client needs, but often reintroducing CPT when they felt the client was ready. As one provider described, “…if we started and they say no, this is too much for me right now, we go back to another generic type of psychotherapy and then when some period of time has gone by, then I would bring it [CPT] back out” (Clinician, private practice).

## Discussion

The purpose of the current study was to qualitatively examine community provider perspectives on initiating CPT with individuals with PTSD and to identify the factors that impact their decision-making. The results presented here reflect data gathered via individual semi-structured interviews with 29 providers during the pre-implementation phase of a larger ongoing longitudinal, mixed method multi-site randomized controlled implementation trial (Wiltsey-Stirman et al., 2017). Our results align with previous literature that has identified factors at the system and provider levels that influence provider decisions in implementing CPT, as well as EBPs more broadly. These findings contribute to extant literature by identifying factors in the decision-making process of providers in community settings on whether to implement CPT.

To date, community-based providers have been an understudied population in the implementation science literature even though they regularly provide care to those with PTSD. Better understanding barriers to delivering EBPs in routine care is of considerable importance, given evidence suggesting CPT implementation in community settings is lower than those found in VA settings (Dondanville et al., 2021a; Finley et al., 2018; Hundt et al., 2018).

Importantly, several of the themes from the present study align with the domains of CFIR. The outer setting refers to factors within the external system of the intervention. In the present study, themes within the outer setting included the role of insurance and the referral source. The inner setting refers to factors within the organization itself, which in the present study included leadership support and time considerations. Lastly, characteristics of individuals can impact implementation. In the present study these included client and provider level factors. Our results suggest the importance of system and organizational level factors to facilitate CPT implementation, as well as the role of client and clinician level factors that impact clinician decision-making in implementation. We further identified intersecting themes and some themes that did not fit neatly within the CFIR framework.

### System and Organization Level Factors

Providers described various system and organizational level factors that influenced their decision to implement CPT, including the role of insurance, leadership support, referral source, and time. Previous research in the VA system has found similar factors that impact the implementation of CPT including fees, scheduling conflicts, and the role of organizational leadership (Lu et al., 2016; Sayer et al., 2017). In the present study, these factors were found to diverge across different settings that providers worked in, i.e., whether in publicly-funded healthcare systems or private practice. Strong leadership in support of EBPs was found to facilitate implementation within publicly funded systems, while, in the private sector, autonomy allowed providers freedom to choose and pursue training in CPT. Across settings, funding for EBPs was a major decision-making factor for whether providers chose to deliver CPT, paralleling the results of previous studies (e.g., Lu et al., 2016).

The theme of referral source was mentioned by clinicians as one that was both a barrier and facilitator of CPT. When referrals were appropriate (i.e., confirmed diagnosis of PTSD), clinicians were more likely to initiate trauma-focused treatment. An important next step for streamlining referral sources was discussed by clinicians as well, who discussed the need for education and advocacy regarding CPT to facilitate appropriate referrals. Larger scale education efforts may be helpful in dispelling myths about trauma and diagnosis of PTSD to referral sources such as Employee Assistance Programs. Another theme that may require additional education for stakeholders is time limitations. This theme emerged across some providers, but not most. It is important that managers, referral sources, and other key stakeholders have sufficient education on the amount of time and preparation that is needed to deliver a trauma-focused intervention to fidelity. This may require clinicians having additional time in their schedules for their clients with PTSD. To increase uptake of CPT with providers, it will be imperative to work with organizational leaders to advocate for sufficient time for providers to engage in training, implementation, and ongoing consultation.

Results of both the present study and the previous literature highlight the importance of adequate insurance coverage within community settings for EBPs. At the most basic level, insurance coverage must allow billing for sufficient sessions to deliver a full dose of CPT. In systems where resources are inadequate or applicable policies do not support CPT implementation, significant system-level changes may be needed to achieve access to EBPs in these settings. This highlights the importance of better allocating system resources to support access to EBPs. The debilitating personal and economic costs associated with PTSD are well documented; additional insurance coverage can go a long way in addressing these significant costs (Jakupcak et al., 2009; Osei-Bonsu et al., 2017; Sayer et al., 2010).

An example of an initiative that addresses mental health needs at a systems level is the Ontario Structured Psychotherapy (OSP) Program. As a publicly-funded initiative in Ontario, Canada, the aim of the OSP Program is to provide evidence-based treatments (EBTs) for depression and anxiety-related problems through a stepped-care model (Antony et al., 2021). CPT is one of the EBPs that is delivered as part of the OSP Program. The OSP Program is premised on the “Improving Access to Psychological Therapies” (IAPT), which has successfully been implemented in England (Clark, 2018). Together, these programs provide examples of systems of care that have sought to increase access to, and enhance the quality of mental health care for large numbers of people. Such initiatives can inform how system resources can be better allocated to support access to trauma-focused EBPs.

### Provider and Client Level Factors

Providers also identified several factors based on their own personal attributes, training background, and sense of self-efficacy that prompted them to learn and implement CPT. Consistent with past literature (Hundt et al., 2017), previous training in CBT was found to be a facilitator of CPT initiation. Further, clinicians' positive perspective of CPT resulted in them advocating for its implementation with clients, organizations, and the broader system (e.g., insurance providers). Providers who were initially skeptical of initiating CPT or manualized approaches reported shifts in their perspective after seeing client gains. This important finding suggests that regardless of providers’ theoretical orientation, buy-in to the utilization of CPT can change over time. It also points to the importance of encouraging clinicians to implement CPT and increase initial motivation to learn new therapies, particularly when skeptical of manualized approaches.

Providers also described client-level factors that influence their decision of whether to implement CPT. Providers reported being more likely to implement CPT based on the following client characteristics: personal motivation; cognitive flexibility; psychosocial stability (e.g., housing, social support system); higher education levels; stable employment; single-trauma; or type of trauma (e.g., sexual assault). Providers noted that they were *less likely* to engage in or offer CPT based on client complexity (e.g., psychosocial challenges like housing issues), significant comorbidities (especially active substance use disorder or personality disorders), or if they presented with multiple traumas or a long-standing trauma history (e.g., childhood sexual abuse). As discussed further below, providers noted that they engaged in shared decision-making based on consideration of several of the above factors. At the same time, it is unclear how such client-level factors influenced genuine client participation in shared decision-making. For example, some providers may have failed to offer CPT as a treatment option in situations of client complexity, comorbidities or long-standing trauma. Future research would benefit from identifying the elements of shared decision-making where such client-level factors are present.

Notably, these factors largely reflect clinical judgement rather than recommendations based on research. Contraindications for delivering CPT, as described by the treatment developers, include imminent suicidality/homicidality, substance use disorder requiring medical detoxification in order to reduce use, and uncontrolled mania or psychosis (Resick et al., 2016). The results of the present study suggest a considerable gap between research and practice for the contraindications of CPT implementation. Therapists in previous literature have also noted these client-level barriers to implementation, including client readiness, comorbidities, and cognitive limitations (Cook et al., 2014; Osei-Bonsu et al., 2017). Further, providers have erroneously voiced concern that “real world” patients will not respond to EBPs as well as patients enrolled in randomized controlled trials (RCTs) with strict eligibility criteria (Foa et al., 2013; McLean & Foa, 2013), despite the growing evidence in effectiveness studies (Turgoose et al., 2018; Watkins et al., 2018). Future research will benefit from further exploring clinician concerns regarding client-level factors. In particular, it will be important to identify if clinician concerns regarding the need for stabilization of complex clients prior to engaging in CPT are based on personal unsuccessful experiences or on misinformation. Relatedly, a low-cost change that may increase CPT uptake at the provider level is to highlight common misconceptions regarding the contraindications for CPT during training workshops.

### Intersecting Factors

Clinicians described a shared decision-making approach, where they involved clients in the process of determining the most fitting therapy to engage in when initially introducing treatment options to their clients. In the current sample, the majority of providers noted that they discussed their treatment approach with their clients at the first session and offered all therapies they were trained in as it was relevant for the client. On occasion, some providers noted that they described all therapies for trauma treatment that they were aware of even if they did not have the training in all of the modalities. If a client opted for a treatment modality that the provider was not trained in, the provider worked with the client to facilitate appropriate referrals. As part of the interview guide, we asked providers “Has anyone refused CPT?” Providers generally noted that clients did not necessarily refuse CPT as they were given a range of options. If they did not select CPT, it was because another therapy felt like a better fit. In the rare instance a client presented with significant psychosocial concerns (e.g., housing issues) providers might introduce CPT but not recommend it as an immediate treatment option.

Although shared decision-making led to less implementation of CPT, this model aligns with current best practices in PTSD care. The 2017 update to the US Department of Veterans Affairs/Department of Defense (VA/DoD, 2017) Clinical Practice Guideline for the Management of PTSD recommends a shared decision-making process for PTSD treatment planning. Further, research has indicated that shared decision-making models improve engagement (Hessinger et al., 2018; Watts et al., 2015) and retention (Mott et al., 2014) in EBPs for PTSD, as well as improvements in PTSD symptoms (Watts et al., 2015). The results of the present study and existing literature suggest that implementation rates may not improve using this model, however, shared decision-making aligns with current policies of client-centered and recovery-oriented care.

At the same time, it is important to recognize that therapist perceptions and preferences may inform this process of shared decision-making. As such biases and misconceptions were not explicitly explored in the current study, future research would benefit from exploring this further.

Our results suggested that community settings are not necessarily responsive to the complexity of client presentations, due to limited resources. Community settings may differ from institutional settings where care is more integrated and thus more responsive to client needs (e.g., stabilization). Clients who have complex psychosocial challenges typically have more integrated care in institutions, which can assist in alleviating these barriers in initiating CPT. The community sample in the present study noted that oftentimes clients will need to be referred elsewhere for these services, leading to a barrier in receiving CPT. At the same time, providers noted that the fragmented community system does not result in never initiating CPT, rather, it may delay when treatment can be started.

### Limitations and Future Directions

The present study has a number of strengths, including the use of a rigorous qualitative methodology grounded in the existing implementation science literature. Additionally, the heterogeneous sample (e.g., providers based in community hospitals and private practices) allowed for results to lend insight into issues of concerns across diverse community settings. Future research can delve further and examine if decision-making processes and factors vary across systems (e.g., hospital setting versus private practice) to better understand patterns that may help explain differences in reach across systems. For example, providers in private practice were more likely to note the challenges of coordinating with private insurance, including limitations placed on the number of sessions, whereas long waitlists and high caseloads were more common in publicly-funded systems. Each of these factors may influence providers’ decisions to engage in CPT and are worth further exploration to ensure CPT’s sustainability over the long-term.

Although the sample was diverse in its range of settings where providers were based, the current study was limited by the small proportion of US-based community clinicians. Future studies would benefit from recruiting more US-based providers to understand unique decision-making factors in both North American contexts. Relatedly, our sample had limited variance in that the majority of providers (53%) identified private practice as their primary work setting. The remainder of the sample worked in a range of settings including (but not limited to), community based mental health and provincial health agencies. Due to the limited number of providers in each setting, we were unable to conduct analysis by type of setting. This is an important area for future research, particularly considering the different constraints that providers in our sample mentioned.

Another limitation is with regard to the demographics of the participant sample. Clinicians in this sample were motivated and had organizational support, as indicated by their participation in a CPT workshop and enrollment in the study looking at consultation. It was noted that at the community level, there is leadership support to attend workshops but there are not always incentives to do so (e.g., in private practice, clinicians attend on their own accord but lose income as a result). Clinicians in the present sample did not view the lack of incentives as a barrier to attend training, but this may represent a biased viewpoint as the current study allowed them to receive training and consultation. Future studies on CPT decision-making within community settings may benefit from interviewing clinicians who are not offered training for their participation in a study.

## Conclusion

This study highlights the complex array of factors that influence the decision-making process of community providers to implement CPT. The current study adds to what is currently a sparse literature on the role of community-provider decision-making to implement CPT, an EBP that has demonstrated the ability to drastically improve the lives and functioning of those with PTSD. Study findings highlight alignment with CFIR domains, consistent with earlier literature in the area examining CPT implementation in community settings (e.g., Marques et al., 2016).

The study is novel in that it is the first to examine providers’ decisions to initiate CPT within community-based organizations, revealing the complex range of factors specifically relevant to these settings. The use of qualitative approaches to assess decision-making processes, facilitated the identification of the complex range of factors that influence therapist decision-making to initiate CPT. Relatedly, this study’s investigation of intersecting factors, or factors that emerged from cross-level analysis was also a novel contribution to the current literature. Such analysis further elucidated nuanced contributions to providers’ decision-making. These findings provide preliminary evidence and give insight into the factors affecting the implementation of an EBP for PTSD in a range of community settings. Study results can inform education and training efforts, preempting concerns cited by community-based clinicians. Specifically, improved understanding of therapist decision-making as elucidated in this study, may help guide future implementation efforts to ensure greater adoption, implementation, and sustainment of CPT in community settings. Finally, given the importance of increasing access to EBPs for PTSD, future studies can build on these findings and elucidate how addressing the intersection of system, provider, and client-level factors may further impact the implementation of CPT in community settings.
